# Supplementary material for: Oxidation-reduction and photophysical properties of isomeric forms of Safranin
Source: PLoS One. 2022 Jun 24;17(6):e0265105. doi: 10.1371/journal.pone.0265105 (PMC9231691; doi:10.1371/journal.pone.0265105)
Supplement: S1 File — (PDF) [file pone.0265105.s002.pdf]

NMR Data Repository§

Oxidation-reduction and photophysical properties of isomeric forms of Safranin

Eskil M.E. Andersen[1], Hsin Wang[2], Joshua S.H. Khoo[3], Jose Cerda[4], and Ronald L. Koder[3,5]*

[1]Department of Biochemistry, The City College of New York, New York, NY 10031

[2]Department of Chemistry, The City College of New York, New York, NY 10031

[3]Department of Physics, The City College of New York, New York, NY 10031

[4]Department of Chemistry, St. Joseph's University, Philadelphia, PA 19131

[5]Graduate Programs of Physics, Biology, Chemistry and Biochemistry, The Graduate Center of CUNY, New York, NY 10016

*Corresponding author

Email: rkoder@ccny.cuny.edu (RK)

§NMR Plots and Assignments were originally done with an atom label scheme that was redone twice. The final conversion key is provided in the next page

New Green

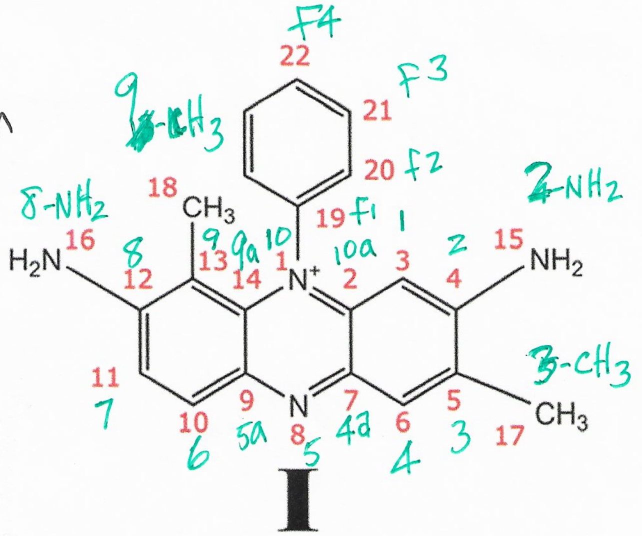

I

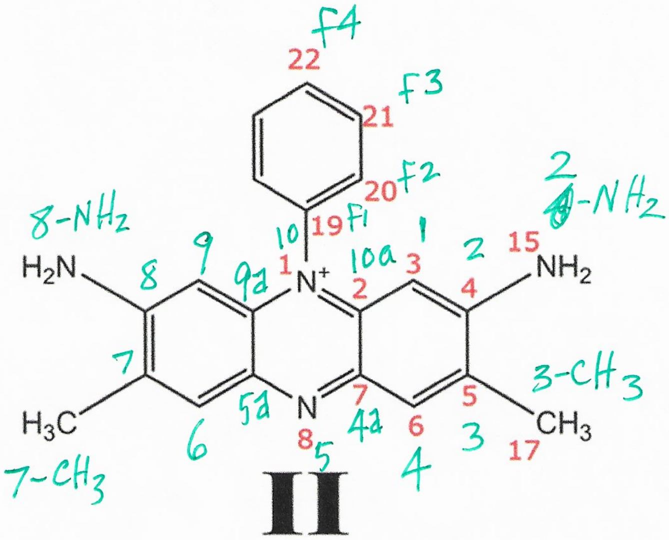

II

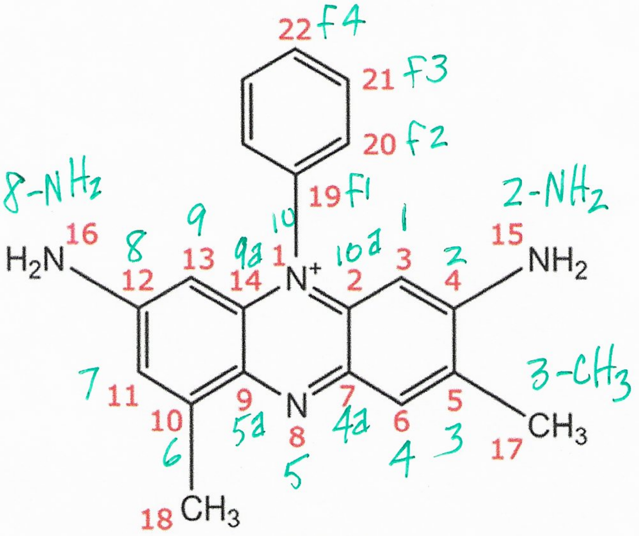

III

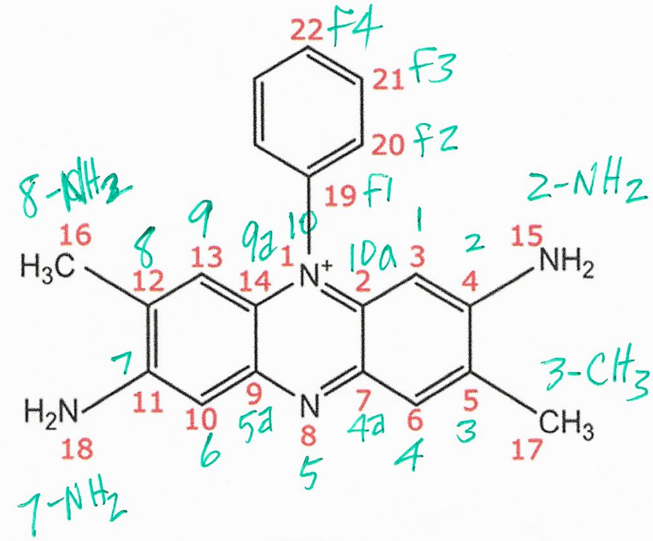

IV

Conversion Key

I:										
1	2	3	4	5	6	7	8	9	10	11
N10+	10a	1	2	3	4	4a	N5	5a	6	7
12	13	14	15	16	17	18	19	20	21	22
8	9	9a	2-NH2	8-NH2	3-CH3	9-CH3	F1	F2	F3	F4
II:										
1	2	3	4	5	6	7	8	9	10	11
N10+	10a	1	2	3	4	4a	N5	5a	6	7
12	13	14	15	16	17	18	19	20	21	22
8	9	9a	2-NH2	8-NH2	3-CH3	7-CH3	F1	F2	F3	F4
III:										
1	2	3	4	5	6	7	8	9	10	11
N10+	10a	1	2	3	4	4a	N5	5a	6	7
12	13	14	15	16	17	18	19	20	21	22
8	9	9a	2-NH2	8-NH2	3-CH3	6-CH3	F1	F2	F3	F4

Compound I

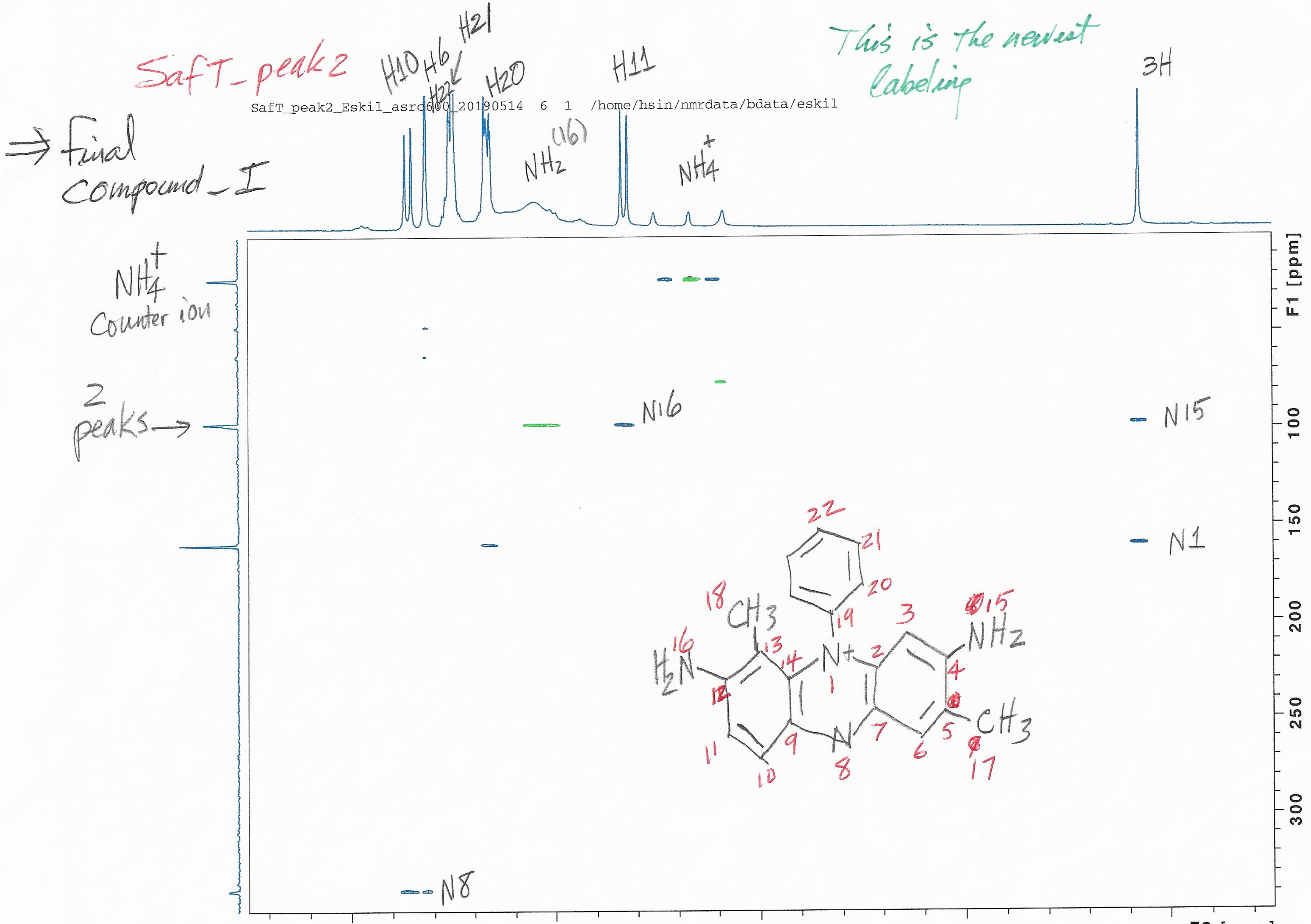
SafT_peak2
⇒ final compound_I
SafT_peak2_Eskil_asrc600_20190514 6 1 /home/hsin/nmrdata/bdata/eskil
H10 H6 H22 H21 H20
NH_2 (16)
H11
NH_4^+
This is the newest labeling
3H
NH_4^+ Counter ion
2 peaks →
N16
N15
N1
N8
F1 [ppm]
F2 [ppm]
8.0
7.5
7.0
6.5
100
150
200
250
300
CH_3
NH_2
H_2N
N^+
N
1 2 3 4 5 6 7 8 9 10 11 12 13 14 15 16 17 18 19 20 21 22

N15-HMBC

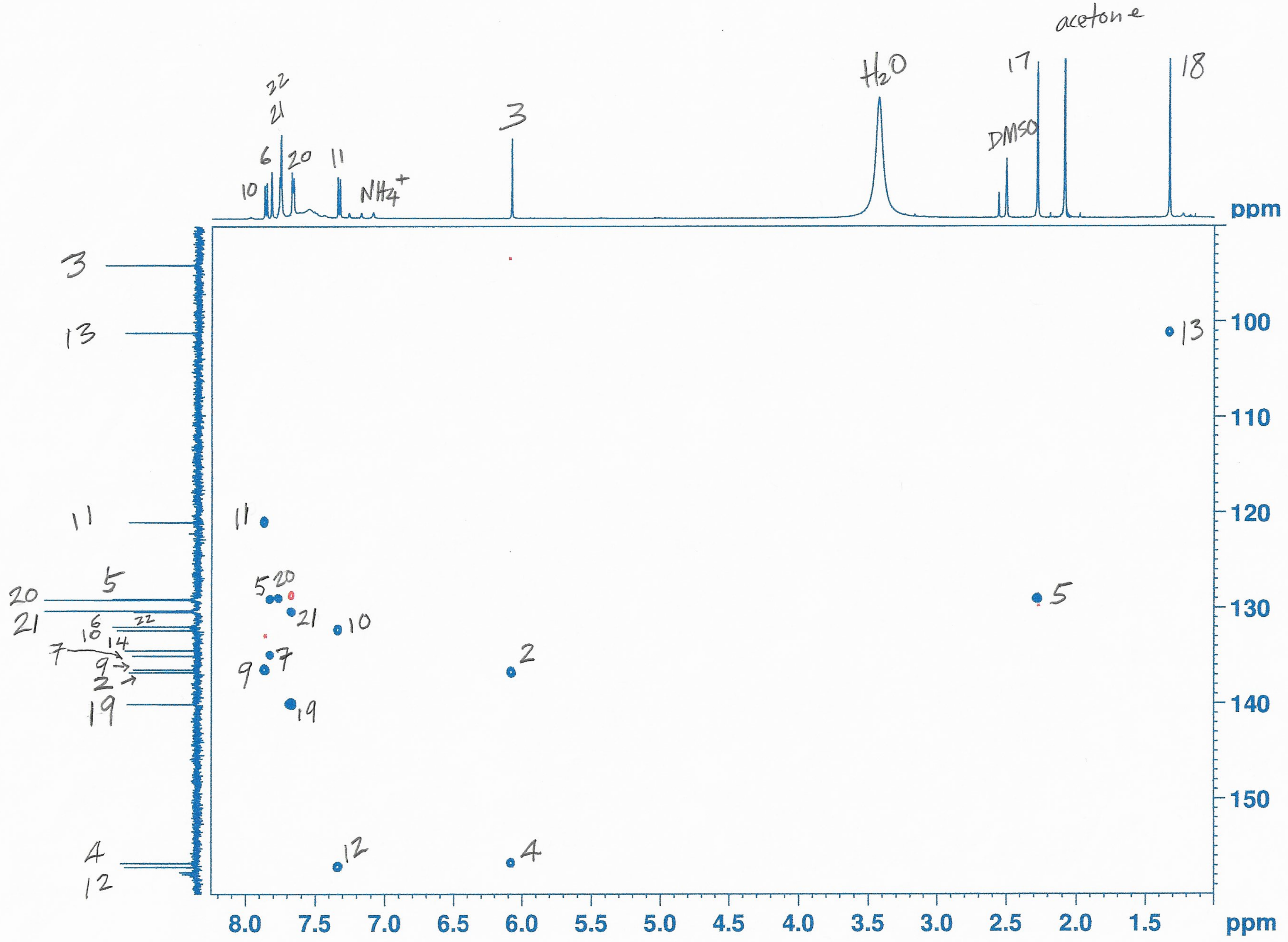

acetone
H2O
DMSO
NH4+
ppm
100
110
120
130
140
150
8.0
7.5
7.0
6.5
6.0
5.5
5.0
4.5
4.0
3.5
3.0
2.5
2.0
1.5
ppm

madq11

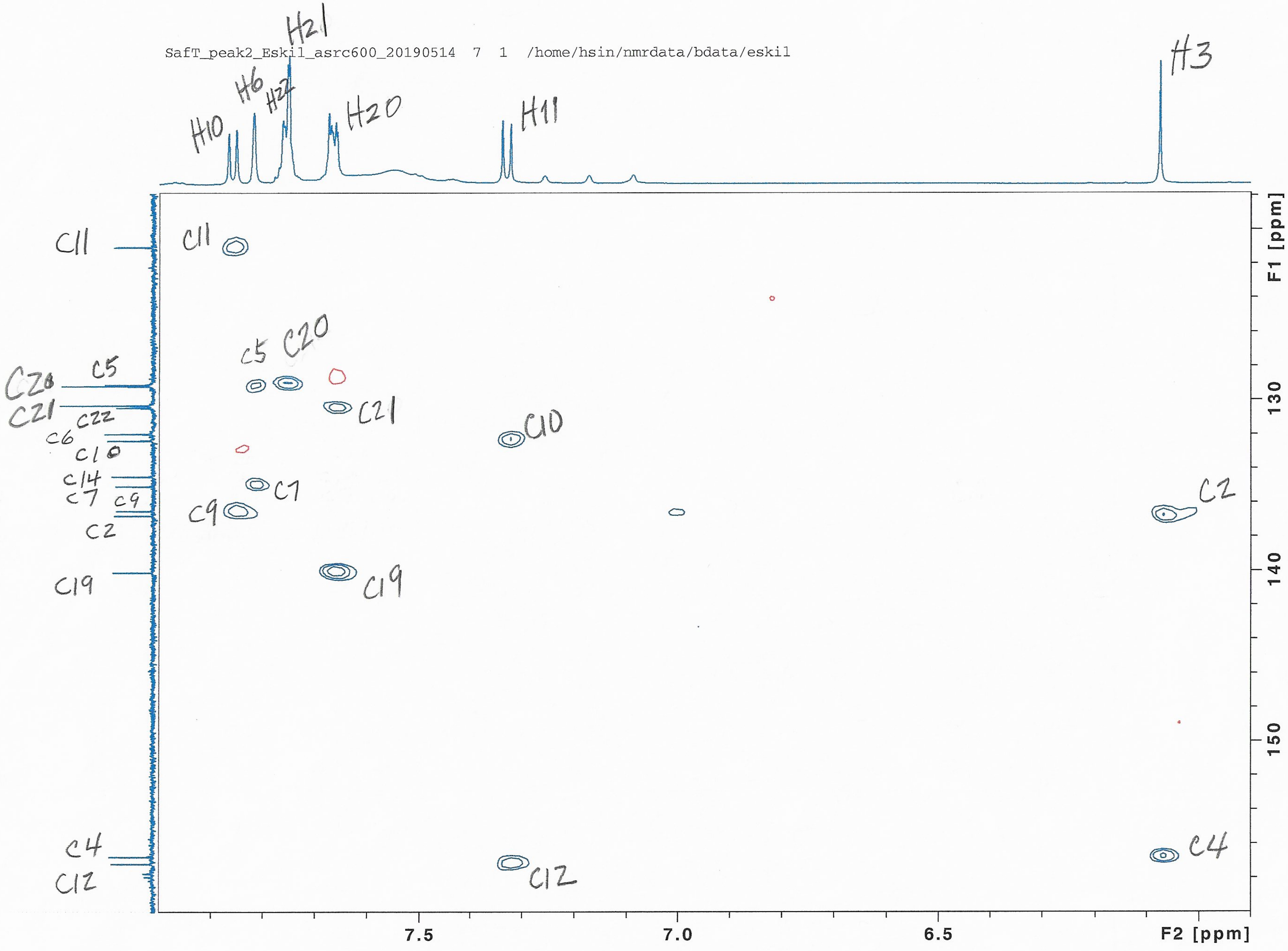

inadeq 11 (region)

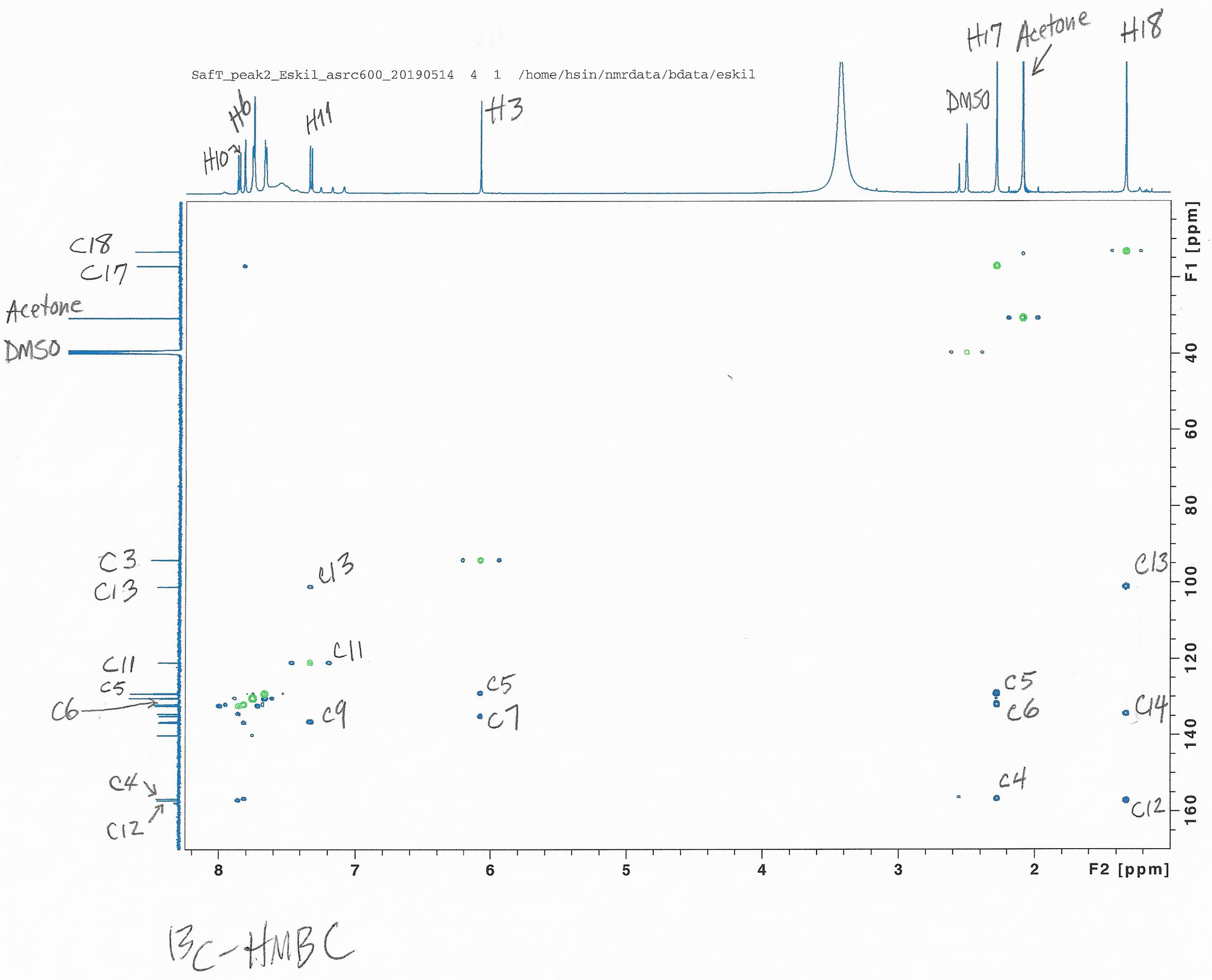
SafT_peak2_Eskil_asrc600_20190514 4 1 /home/hsin/nmrdata/bdata/eskil
H10
H6
H11
H3
DMSO
H17
Acetone
H18
C18
C17
Acetone
DMSO
C3
C13
C11
C5
C6
C4
C12
C13
C11
C9
C5
C7
C6
C14
F1 [ppm]
F2 [ppm]
¹³C-HMBC

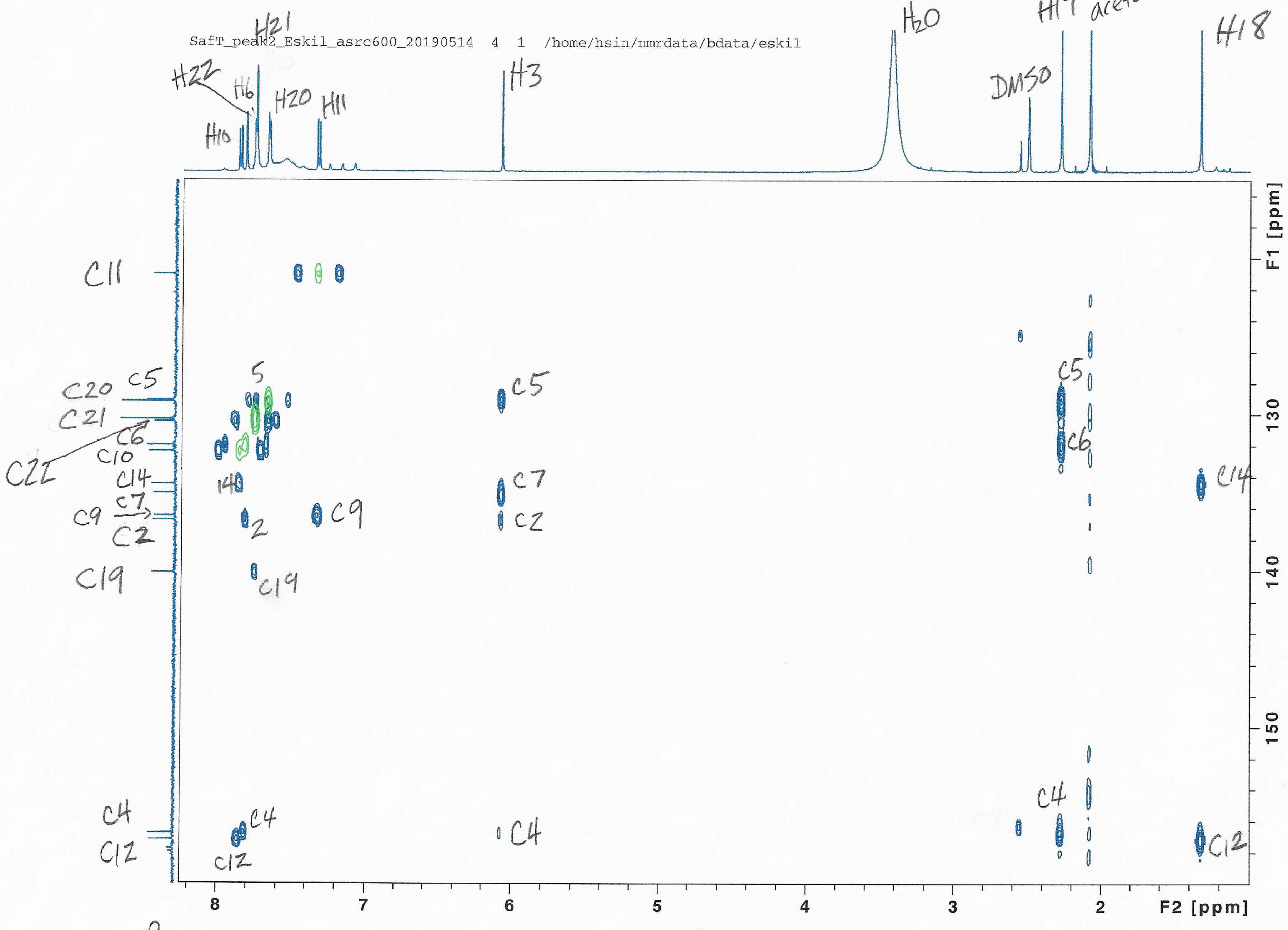

Overlay HSQC(green) HMBC(blue) ^{13}C

SAFT_peak2 H1 assignment

Peak	ν(F1) [ppm]	Intensity [abs]	Annotation
1	7.8662	108341.04	}
2	7.8510	116460.55	} 7.858, d, H10
3	7.8170	152940.91	s, H6
4	7.7599	135634.41	}
5	7.7570	124597.31	} 7.7~~58~~6, m, H22
6	7.7511	256309.37	} 7.75, m, ~~H20~~ H21
7	7.7485	274413.30	}
8	7.6727	151673.62	}
9	7.6684	126621.58	} 7.67, m, ~~H21~~ H20
10	7.6659	111807.34	}
11	7.6598	132438.75	}
12	7.6571	112099.86	}
13	7.5489	31242.37	broad, NH_2
14	7.3361	136008.72	}
15	7.3209	129056.82	} 7.328, d, H11
16	7.1702	18784.16	t, NH_4^+
17	6.0749	261753.79	s, H3
18	2.2762	509744.15	s, H17 (CH_3)
19	1.3259	652921.70	s, H18 (CH_3)

N15 assignments (From N15-HMBC)

N1 160.1 ppm

~~N15~~ N8 339.3 ppm

N15 97.2 ppm

N16 98.0 ppm

NH_4^+ counter ion 22.7 ppm

SAFT_peak2 C13 assignment

Peak	ν(F1) [ppm]	Intensity [abs]	Annotation
1	206.5402	96545.38	acetone
2	157.7166	20370.16	TFE-COO
3	157.1470	96684.19	C12
4	156.7353	102063.88	C4
5	140.0667	93138.53	C19
6	136.7409	90213.75	C2
7	136.4524	84922.62	C9
8	135.0040	86223.09	C7
9	134.4387	96492.12	C14
10	132.3304	106964.50	C10
11	131.9459	113155.00	C6
12	130.4178	83802.72	C22
13	130.2843	224186.88	~~C20~~ C21
14	129.1346	219948.84	~~C21~~ C20
15	129.0596	112665.56	C5
16	120.9981	90892.34	C11
17	101.1461	96349.72	C13
18	94.0638	123458.56	C3
19	30.6949	600818.94	acetone
20	17.0508	193480.66	C17
21	13.2311	200940.38	C18

Compound II

SafT_peak3 Symmetric ⟹ Final Compound_II

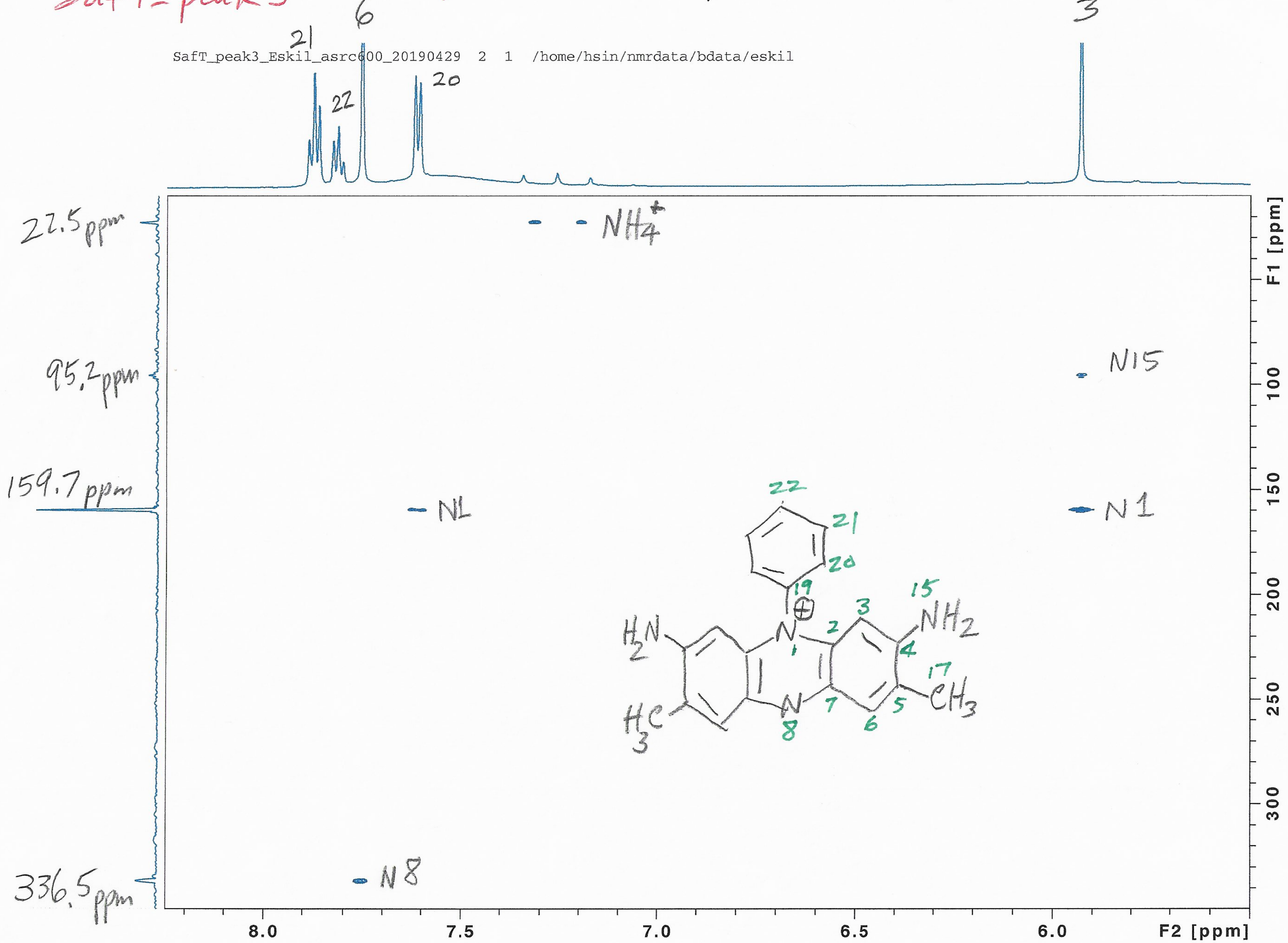

N15-HMBC

$C_{18}H_{19}N_4$

SafT_peak3

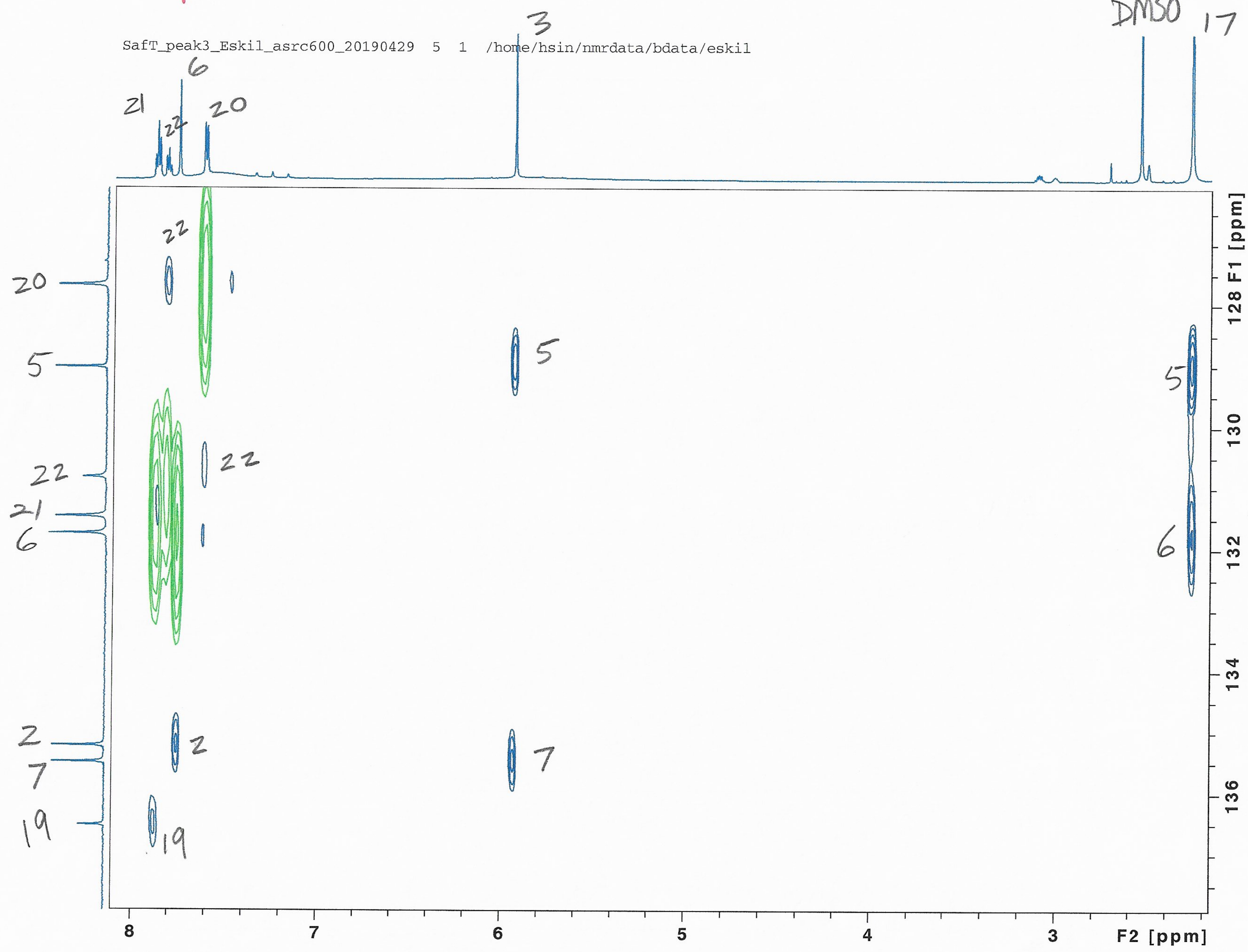

C13- HSQC (green), HMBC (blue) — region

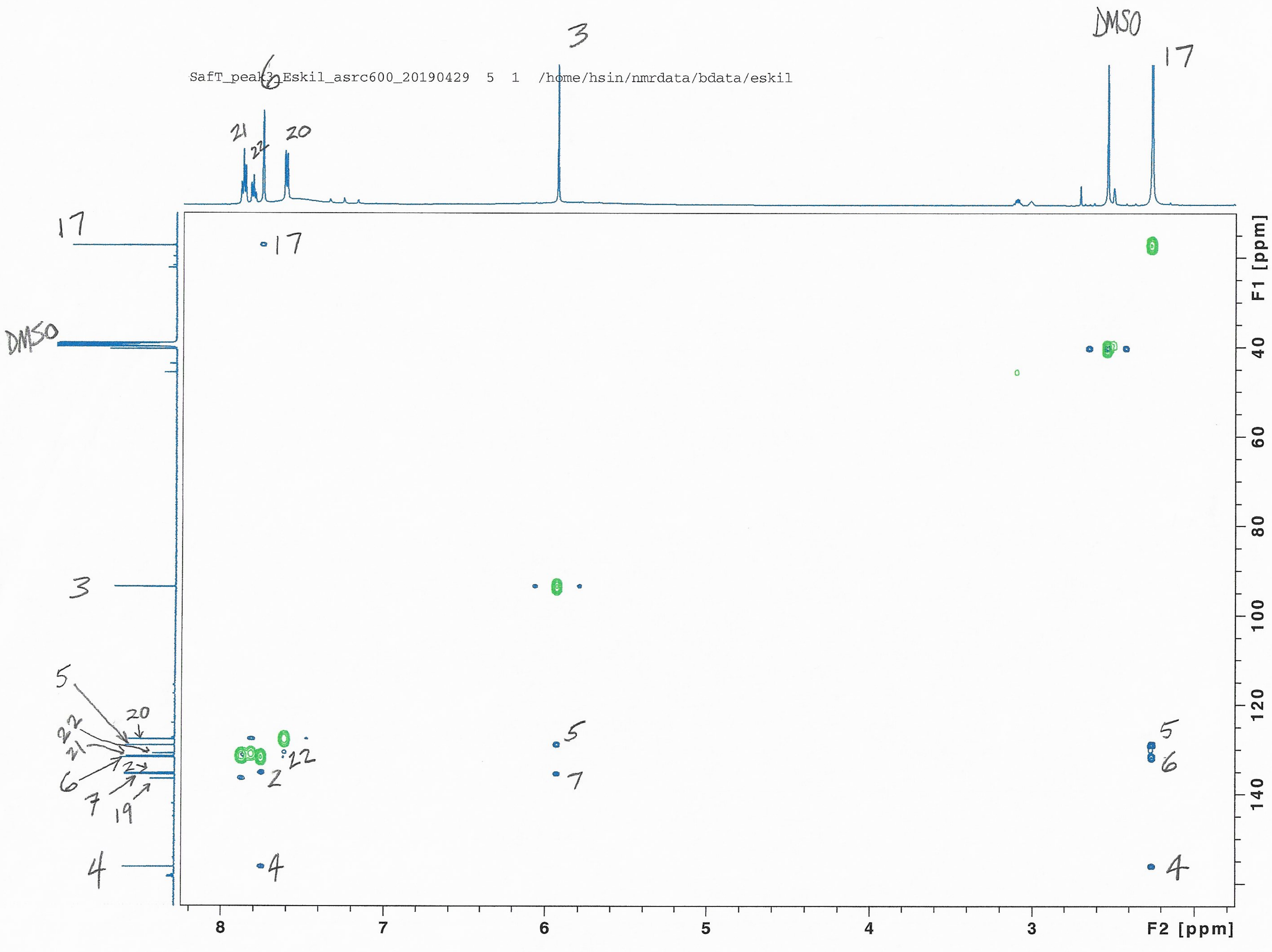

C13-HSQC (green), HMBC (blue)

Peak	v(F1) [ppm]	Intensity [abs]	Annotation	
1	158.3686	102167.34	CF3COO-	} q
2	158.1409	98418.94	CF3COO-	
3	156.2150	731377.09	C4	
4	136.4990	339893.06	C19	
5	135.4654	706146.88	C7	
6	135.2018	707764.28	C2	
7	131.7268	772086.00	C6	
8	131.4480	681200.66	C21	
9	130.8025	307922.97	C22	
10	129.0038	692971.59	C5	
11	127.6577	652229.00	C20	
12	117.4134	30232.59	CF3	} q
13	115.4503	27416.16	CF3	
14	93.5243	865726.31	C3	
15	17.2413	1460289.72	C17	

SafT_peak 3 symmetric

Peak	v(F1) [ppm]	Intensity [abs]	Annotation
1	7.8743	1140833.05	H21, t
2	7.8141	609189.80	H22, t
3	7.7544	1945320.17	H6, s
4	~~7.6183~~	1111930.53	H20 } d, 7.612
5	~~7.6056~~	1051149.59	H20 }
6	7.2567	140791.42	NH4+, t (from ^{14}N)
7	5.9297	2875198.02	H3, s
8	2.2616	7150317.56	H17, s

N15HMBC

N15 95.2ppm

N1 159.7ppm

N8 336.5ppm

NH_4^+ 22.5ppm

Compound III

SaFT_peak5 Translation key   Final ⇒ compound III   New labeling 20190520 (green)

1	2	3	4	5	6	7	8	9	10	11	12	13	14	15	16	17	18	19	20	21	22
~~1~~	~~2~~	~~3~~	~~4~~	~~16~~	~~11~~																
1	14	13	12	16	11	10	18	9	8	7	6	5	17	4	15	3	2	no change			

This translation key is not the final version

This compound was initially named 3 then 5 and back to III

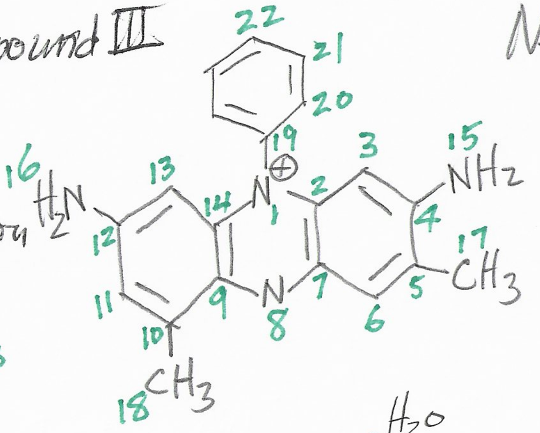

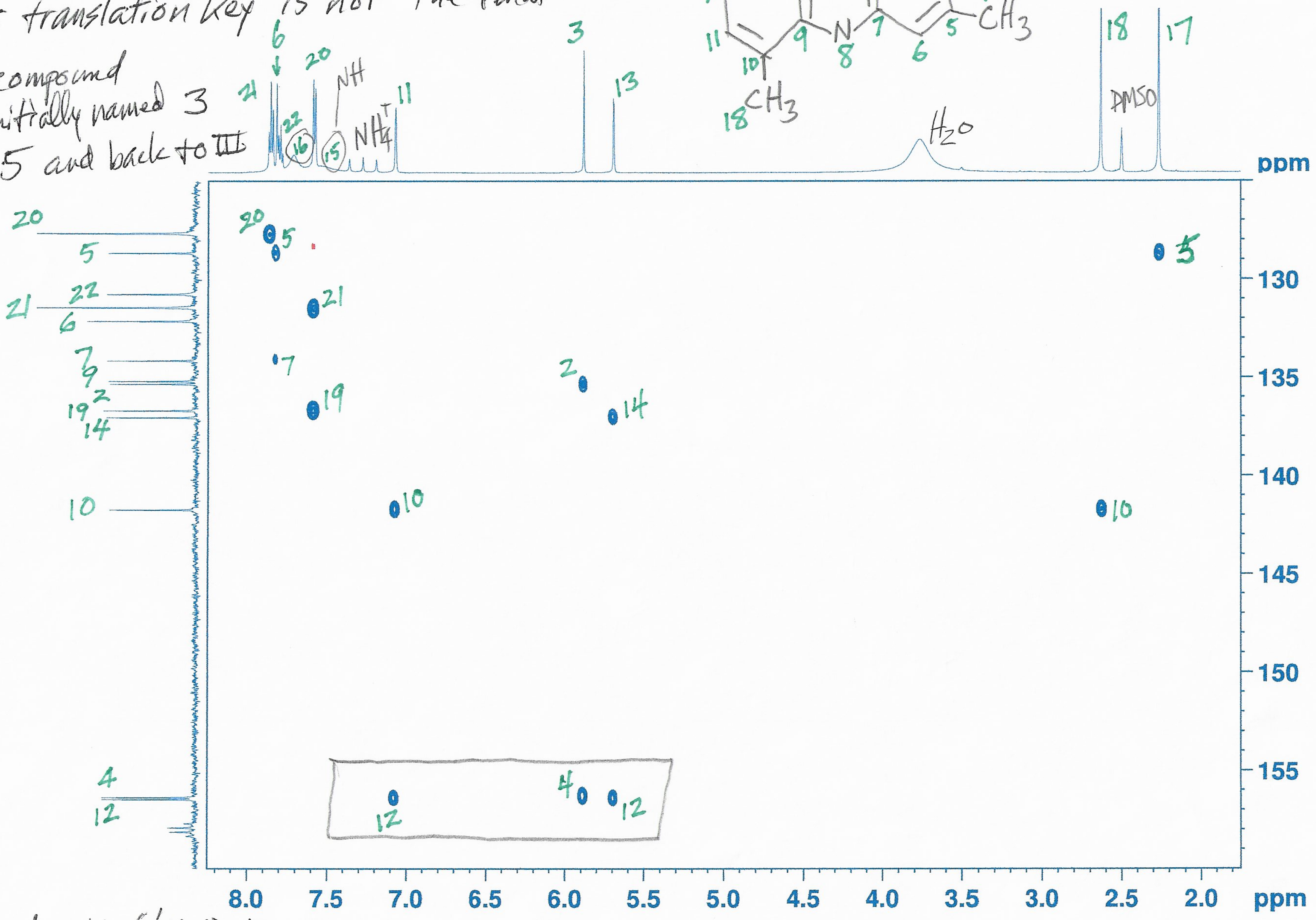

inadeq11 (^1H, ^{13}C)

This Component came out last in HPLC, i.e. least polar

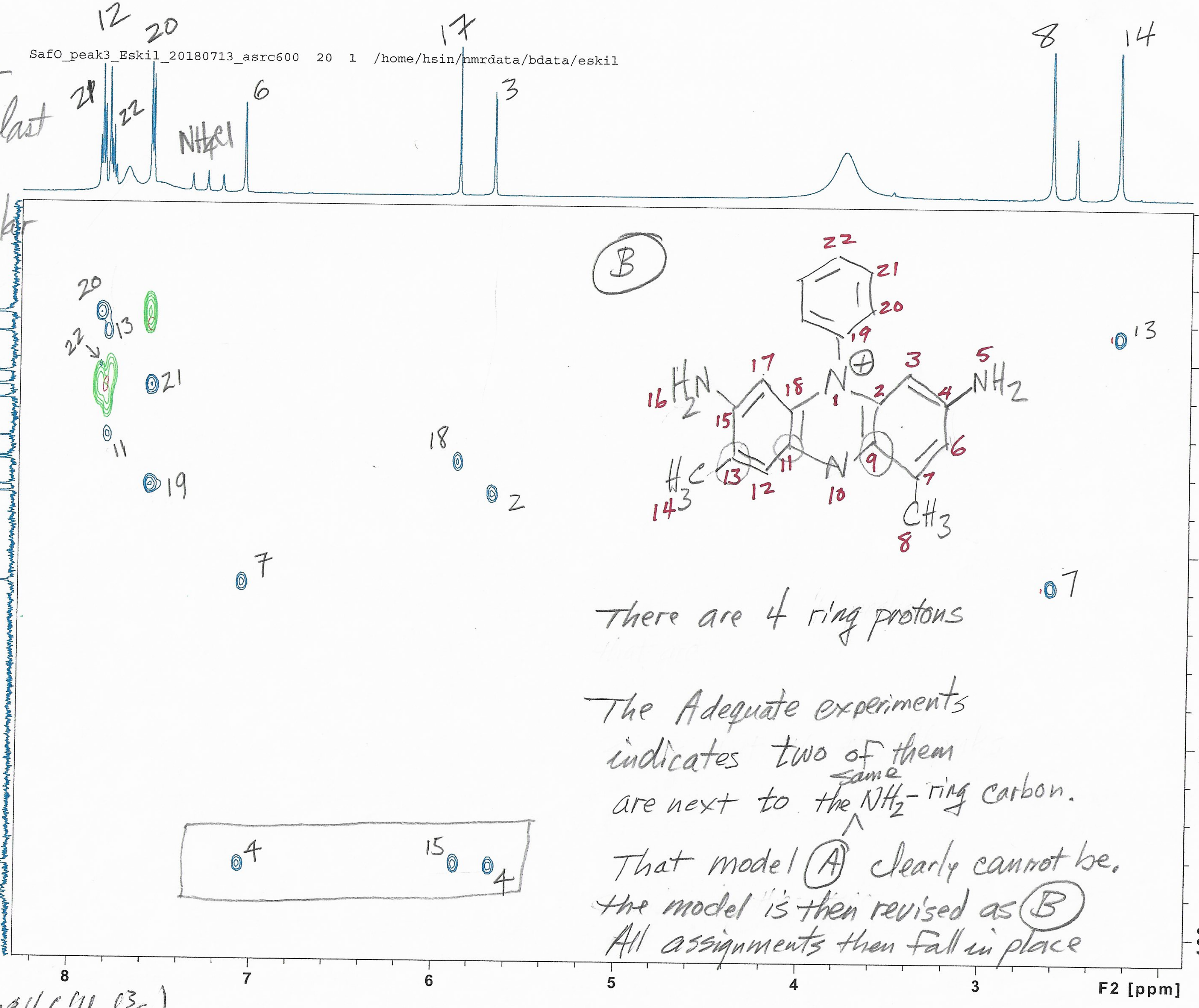

inadeq11 (^1H, ^{13}C)

SafO_peak5 adeq1n vs adeq11 (green) (blue & red)

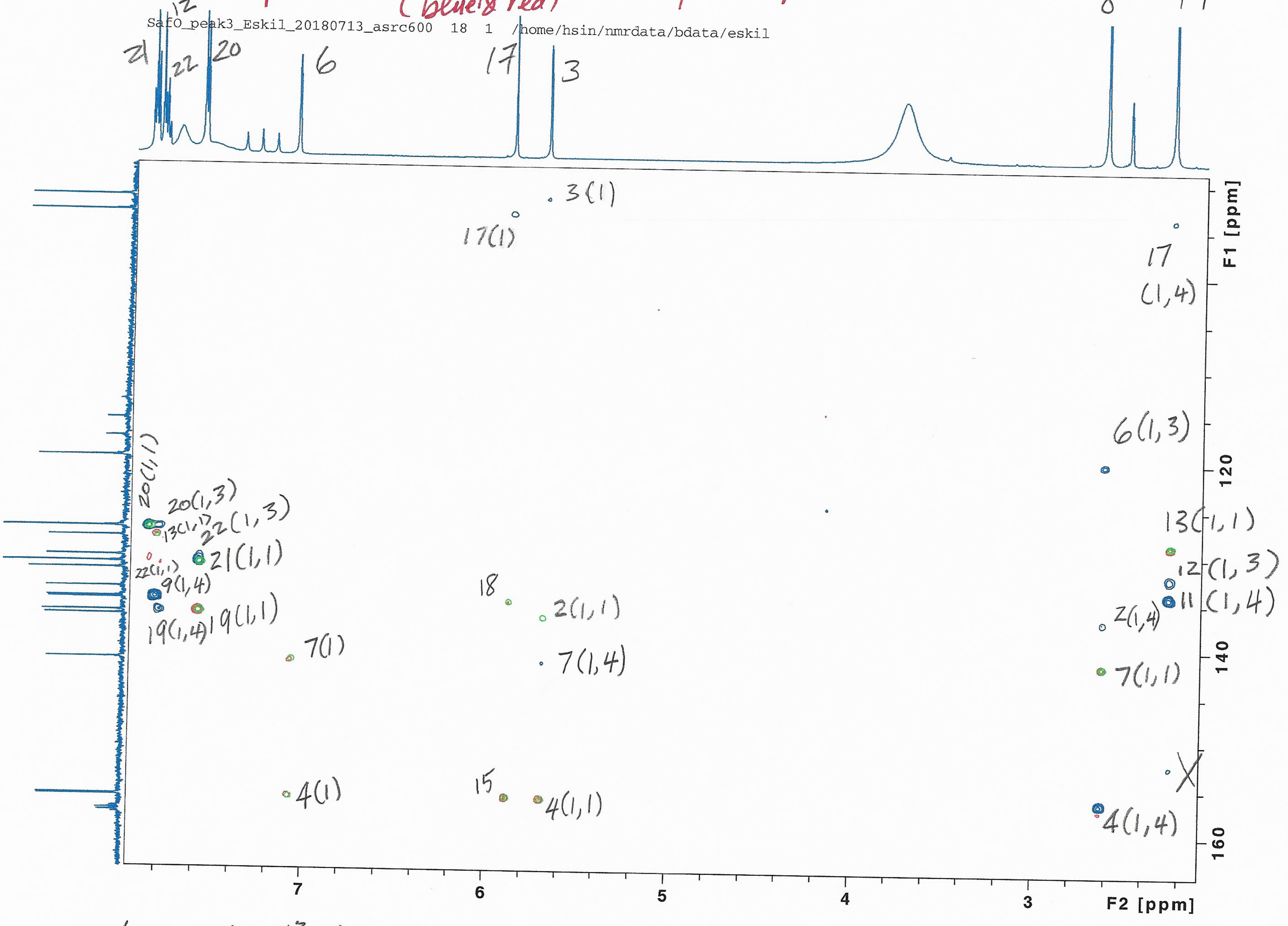

adeq1n (^1H, ^{13}C)

SafT_peak5

SafO_peak3_Eskil_20180713_asrc600   2   1   /home/hsin/nmrdata/bdata/eskil

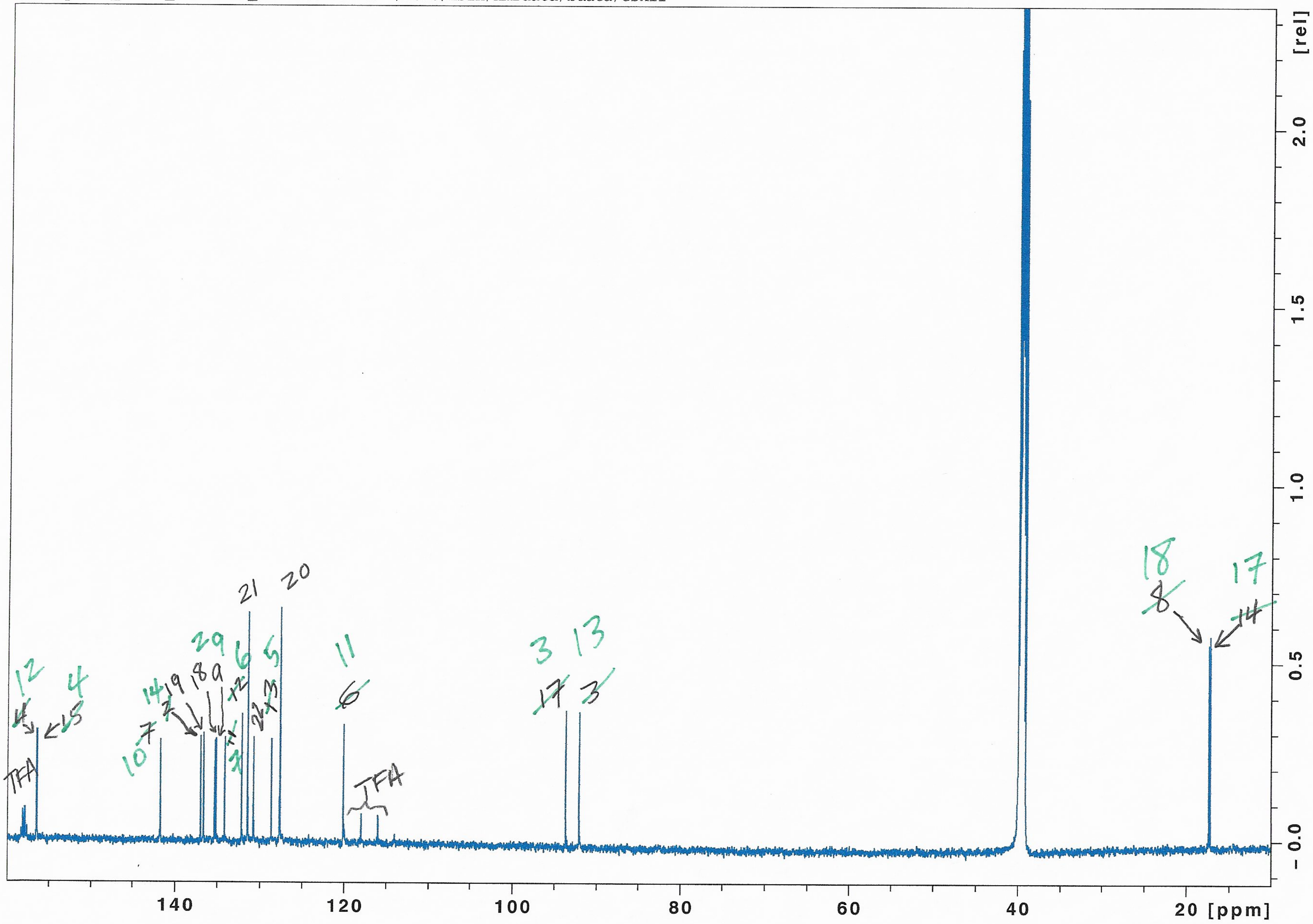

13C

SafT-peak 5

SafO_peak3_Eskil_20180713_asrc600 2 1 /home/hsin/nmrdata/bdata/eskil

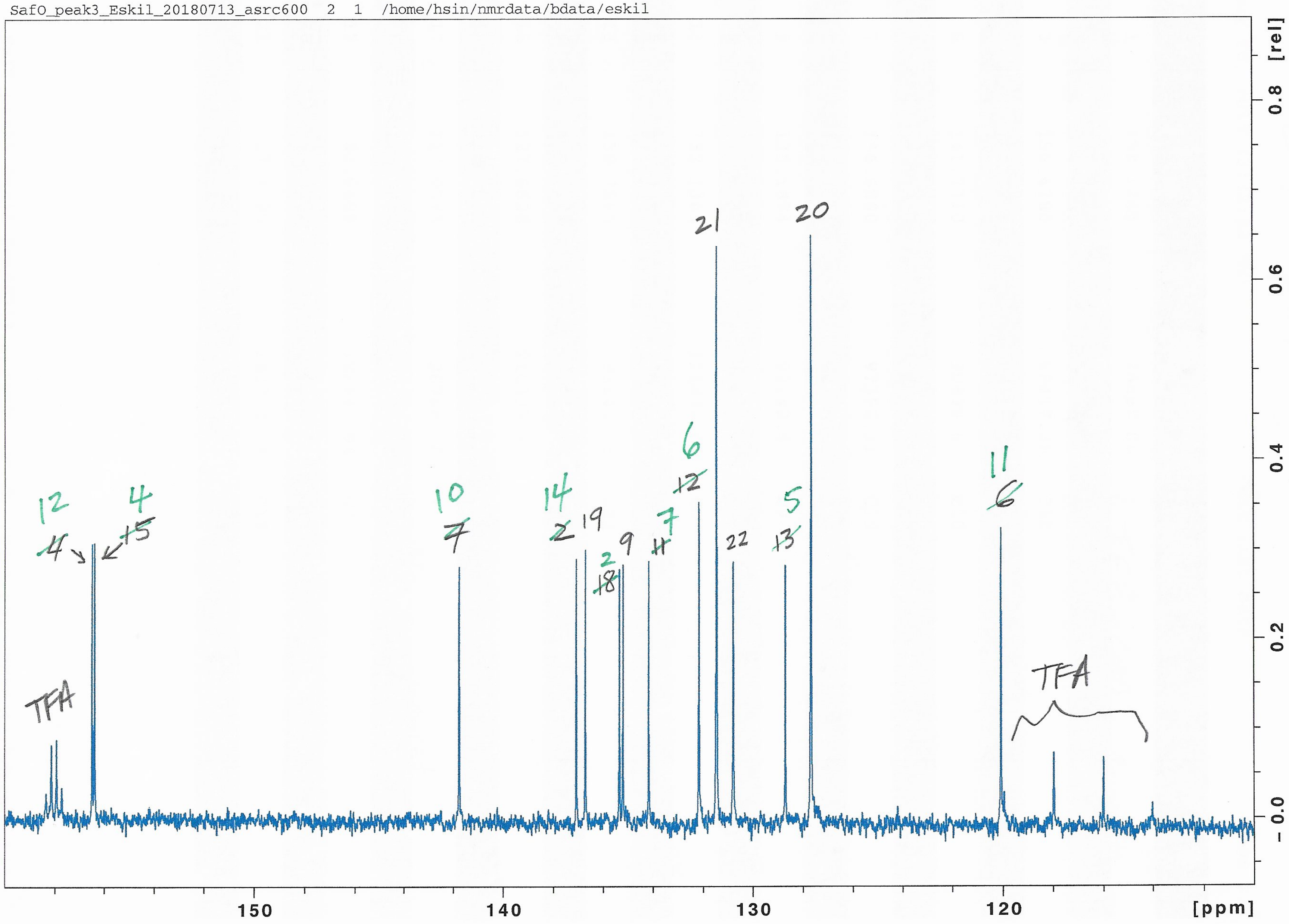

^{13}C (region)

The starting model was thought to be asymmetric

Renamed to SafT_peak 5

model (A)

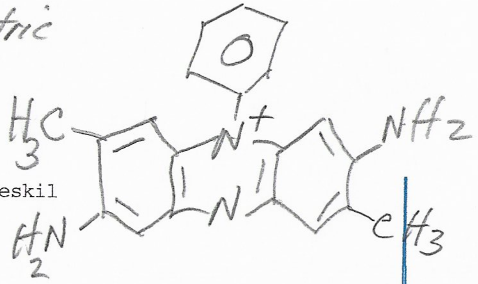

However, this N15-HMBC shows that 2 of H1 can be connected to the N^+ and NH_2

So it seems to be inconsistent

N15-HMBC

SafO_peak3_Eskil_20180713_asrc600 5 1 /home/hsin/nmrdata/bdata/eskil

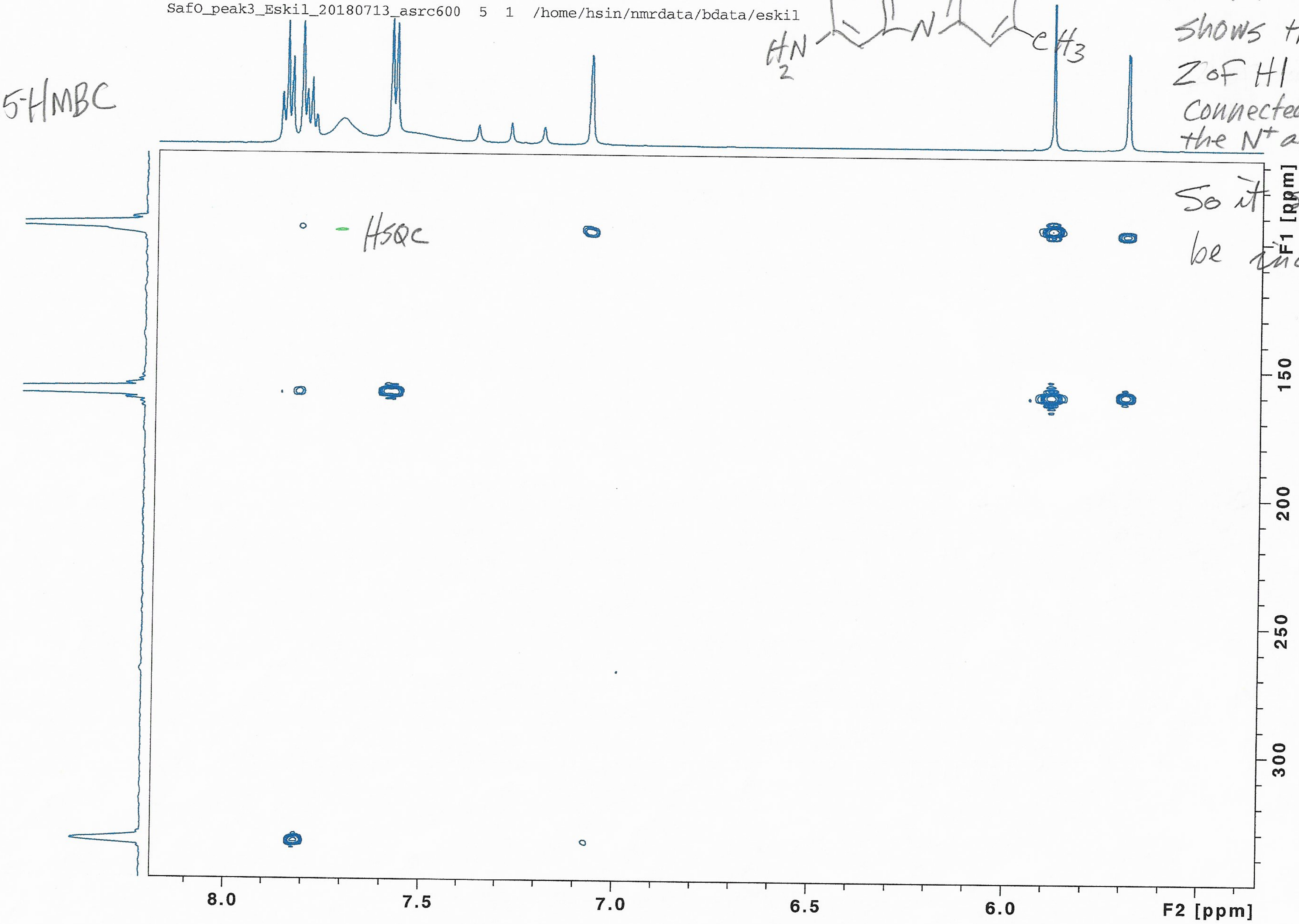

SaFT_peak5

green is new labeling

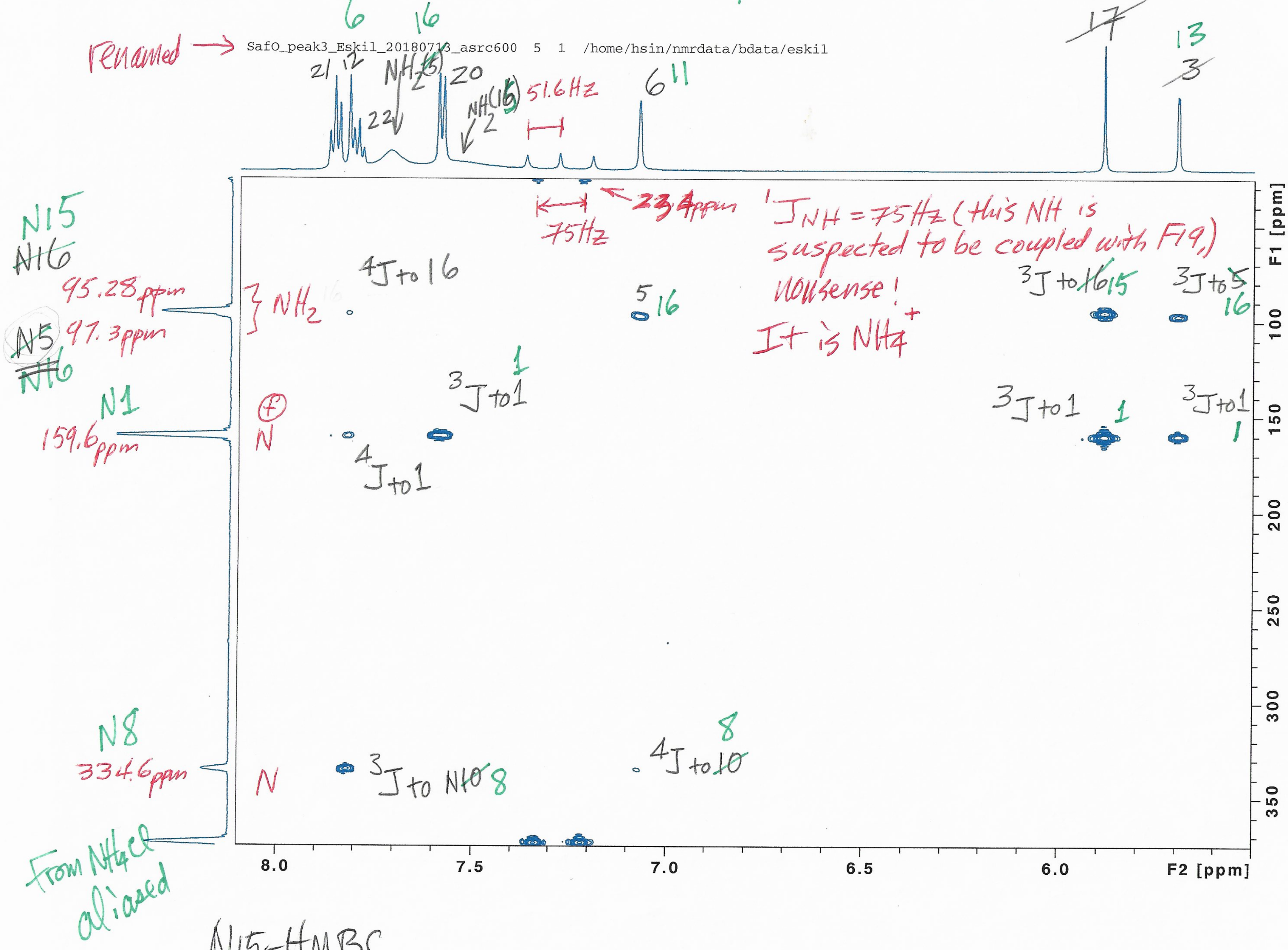

N15-HMBC

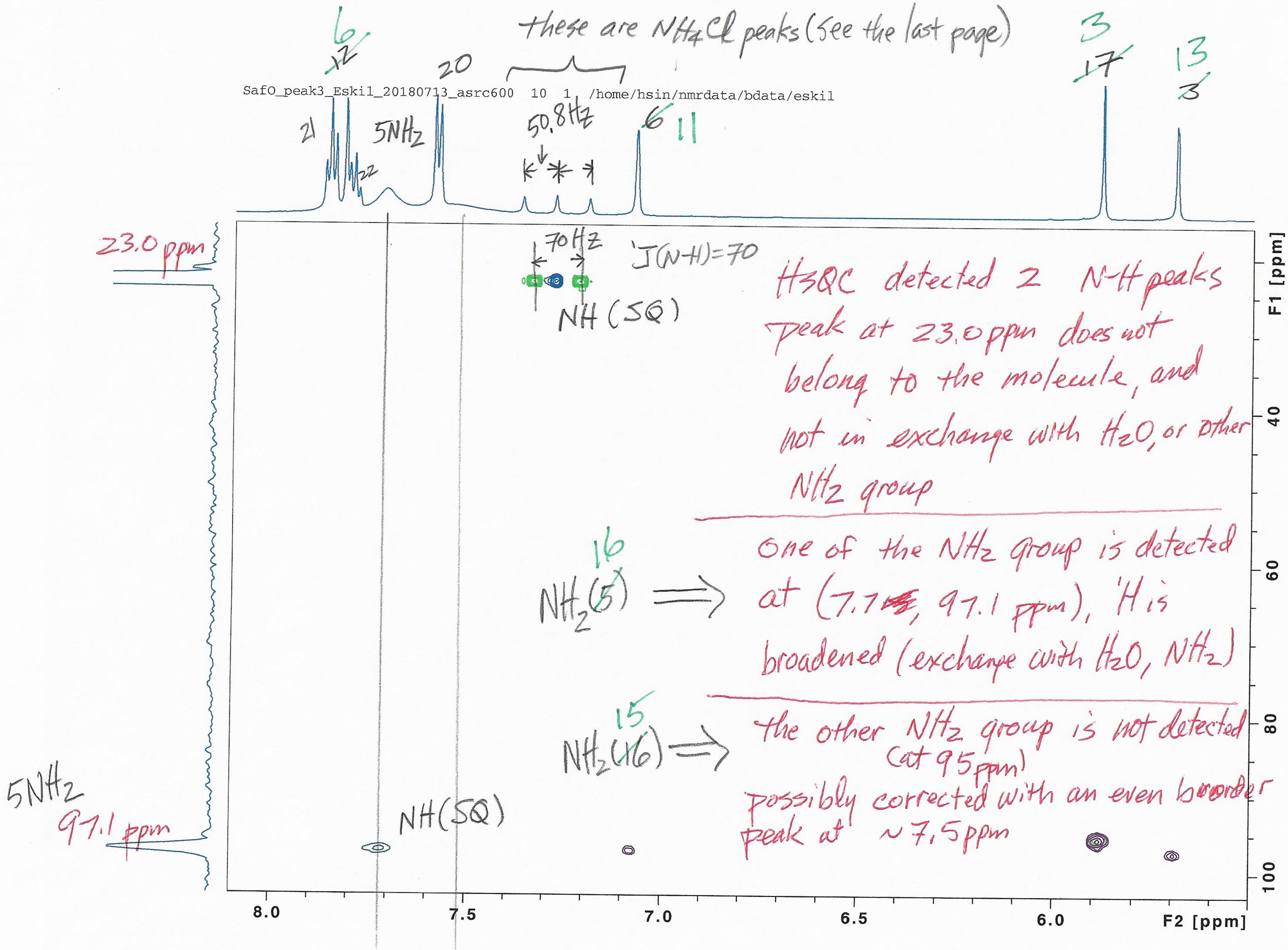
these are NH_4Cl peaks (see the last page)
SafO_peak3_Eskil_20180713_asrc600 10 1 /home/hsin/nmrdata/bdata/eskil
21
5NH_2
22
20
50.8Hz
6 11
3 17
13 3
70Hz
^1J(N-H)=70
NH (SQ)
HSQC detected 2 N-H peaks
peak at 23.0ppm does not belong to the molecule, and not in exchange with H_2O, or other NH_2 group
One of the NH_2 group is detected at (7.7, 97.1 ppm), ^1H is broadened (exchange with H_2O, NH_2)
NH_2(5) 16
the other NH_2 group is not detected (at 95ppm)
possibly corrected with an even broader peak at ~7.5ppm
NH_2(16) 15
23.0 ppm
5NH_2
97.1 ppm
NH(SQ)
F1 [ppm]
F2 [ppm]

N15-HSQC

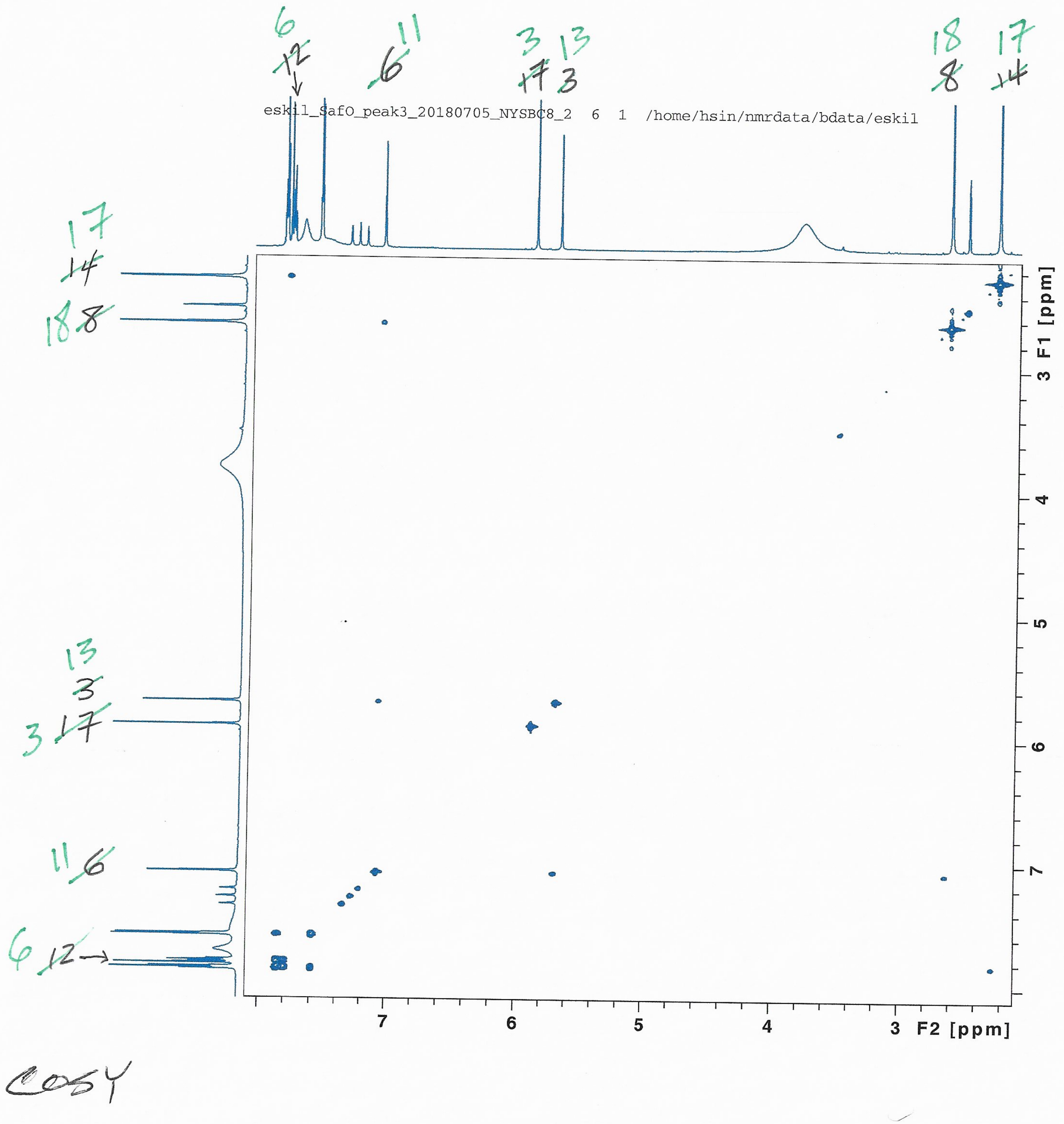
eskil_SafO_peak3_20180705_NYSBC8_2 6 1 /home/hsin/nmrdata/bdata/eskil
F1 [ppm]
F2 [ppm]
COSY

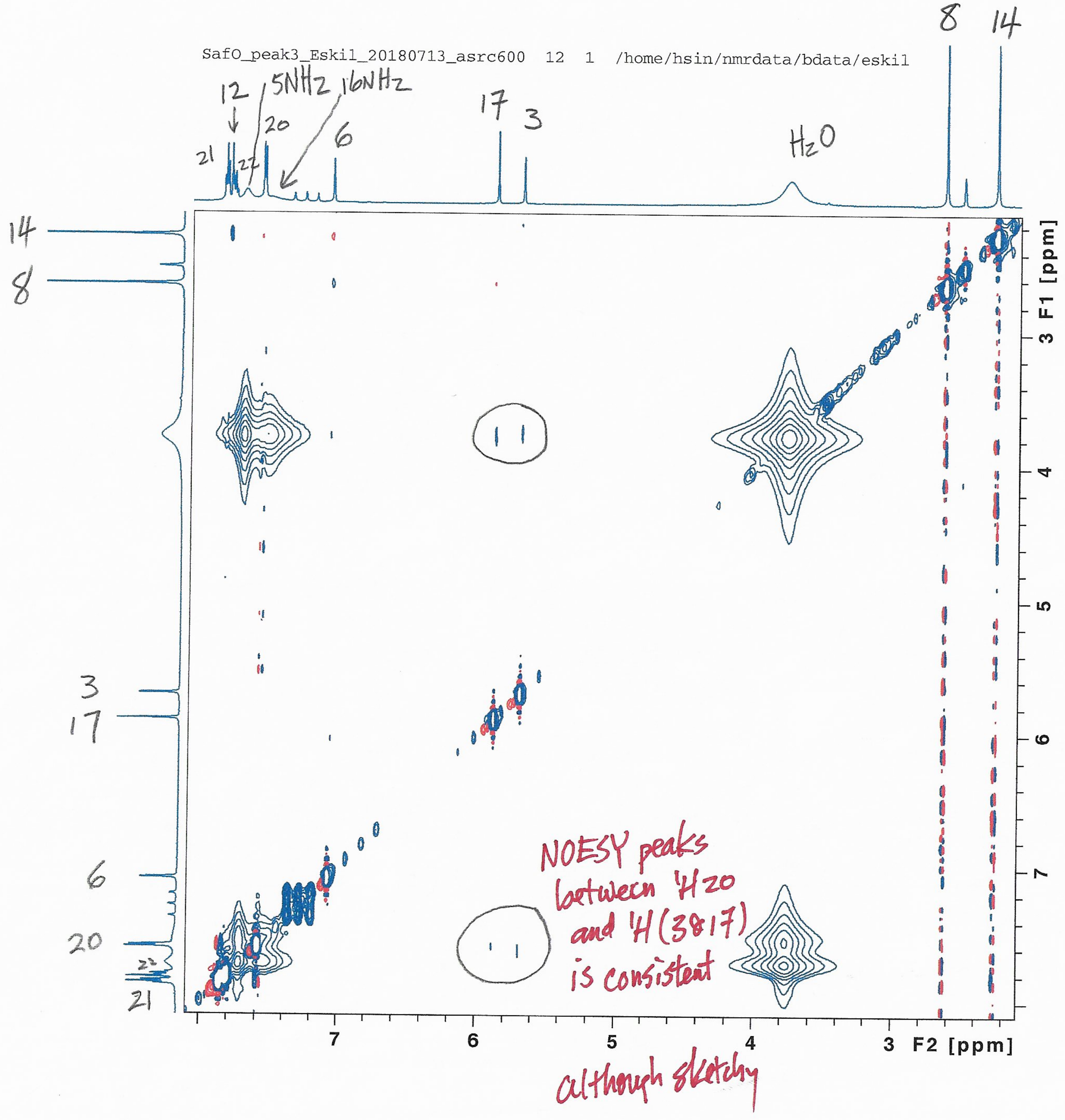
SafO_peak3_Eskil_20180713_asrc600 12 1 /home/hsin/nmrdata/bdata/eskil
8
14
12
5NH2 16NH2
20
21
22
6
17
3
H2O
3
17
6
20
F1 [ppm]
F2 [ppm]
NOESY peaks between 1H2O and 1H(3&17) is consistent
although sketchy

SafO_peak3_Eskil_20180713_asrc600 11 1 /home/hsin/nmrdata/bdata/eskil

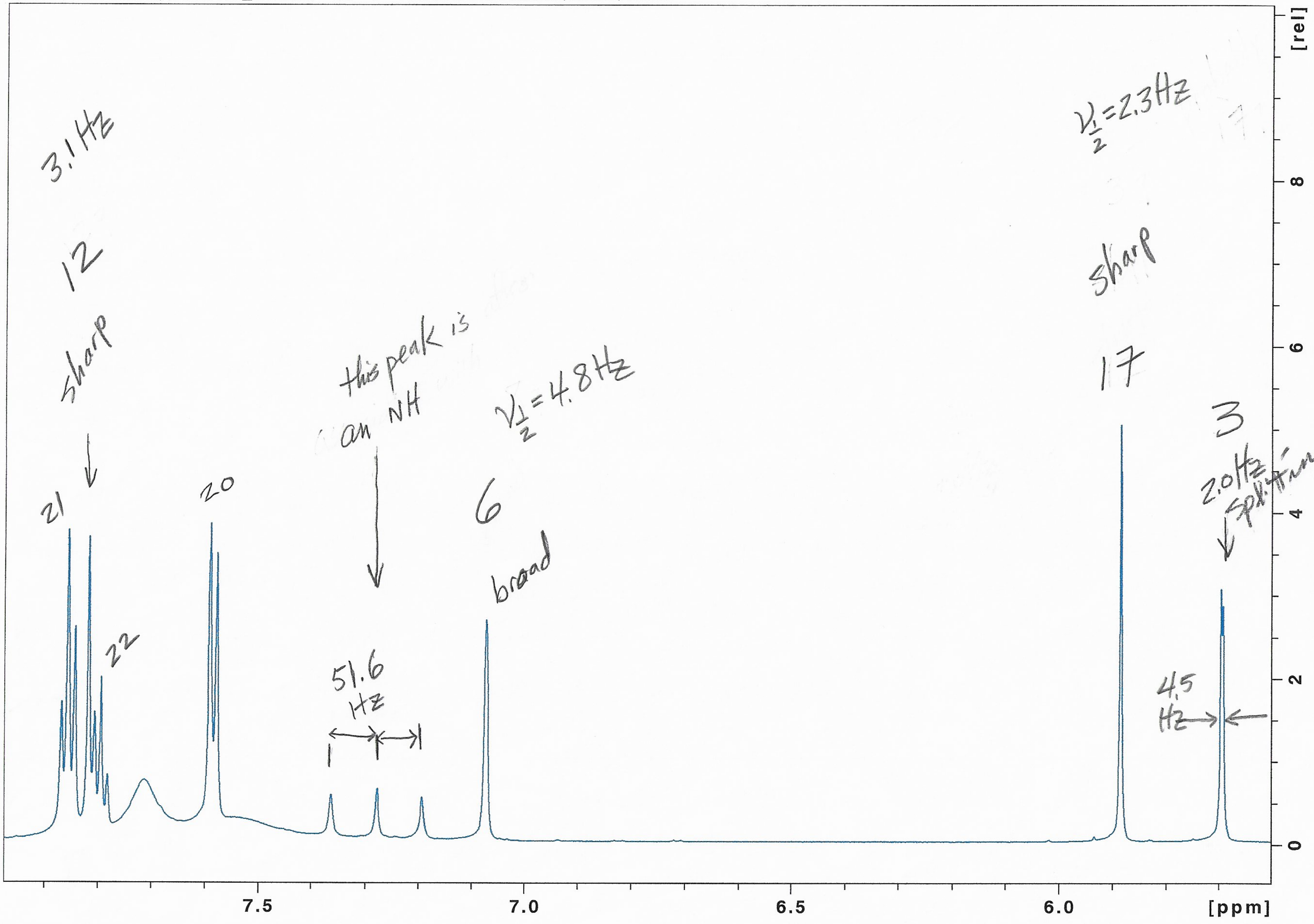

^1H (C region)

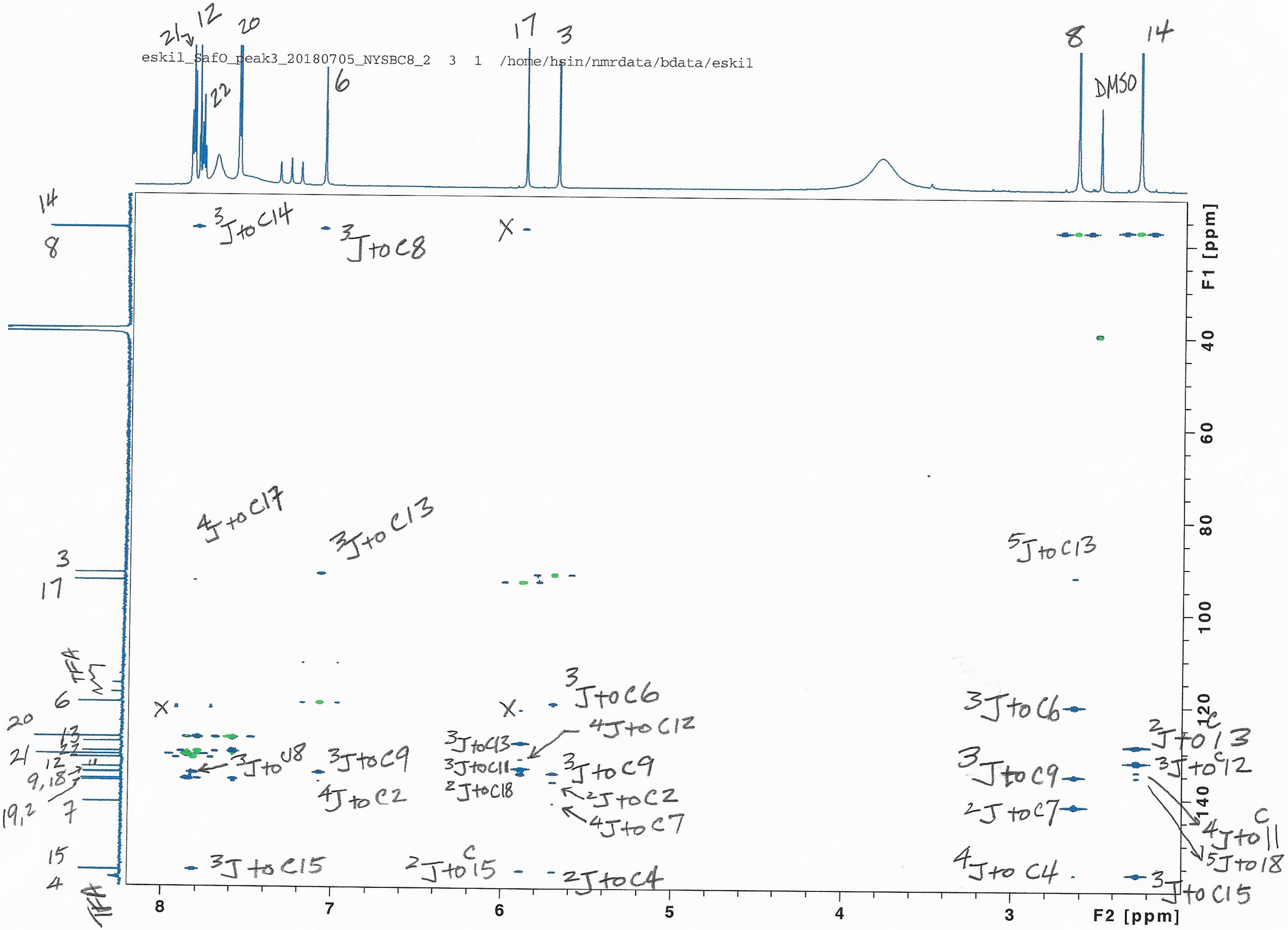
eskil_SafO_peak3_20180705_NYSBC8_2 3 1 /home/hsin/nmrdata/bdata/eskil
F1 [ppm]
F2 [ppm]
DMSO
TFA
³J to C14
³J to C8
⁴J to C17
³J to C13
⁵J to C13
³J to C6
³J to C6
⁴J to C12
³J to C13
³J to C11
²J to C18
³J to C9
²J to C2
⁴J to C7
³J to C9
⁴J to C2
³J to C9
²J to C7
³J to C15
²J to C15
²J to C4
⁴J to C4
³J to C15
²J to C13
³J to C12
⁴J to C11
⁵J to C18
HMBC (¹³C)
