# Supplementary material for: Oxidation-reduction and photophysical properties of isomeric forms of Safranin
Source: PLoS One. 2022 Jun 24;17(6):e0265105. doi: 10.1371/journal.pone.0265105 (PMC9231691; doi:10.1371/journal.pone.0265105)

The City College of New York

May 6th, 2022

Department of Physics

RONALD L. KODER
JAMES L. PEACE CHAIR
DEPARTMENT OF PHYSICS
419 MARSHAK SCIENCE BLDG.
THE CITY COLLEGE OF NEW YORK
160 CONVENT AVENUE
NEW YORK, NY 10031
212-650-5583 (OFFICE)
RKODER@CCNY.CUNY.EDU

Dear Dr. Niederman,

Please find attached a re-revised version of our manuscript “Oxidation-reduction and photophysical properties of isomeric forms of Safranin”, PONE-D-22-05311. We have incorporated changes that address all the comments brought forth by both reviewers, and we believe the paper is greatly improved as a result of these suggestions. We thank the reviewers first for these suggestions, and second for responding so quickly.

A detailed list of the reviewer comments and our responses follows, with their text in black and our responses in green:

Journal Requirements:

(1) Please ensure that your manuscript meets PLOS ONE's style requirements.

We have reformatted the manuscript to fit the Journal’s style guide.

(2) However, funding information should not appear in the Acknowledgments section or other areas of your manuscript. We will only publish funding information present in the Funding Statement section of the online submission form. Please remove any funding-related text from the manuscript and let us know how you would like to update your Funding Statement.

We have removed the Acknowledgements section of the text completely. However, we were recently reminded that we should also acknowledge the funding that supported the NMR spectrometers used in the paper. As such, the we have added NIH grants S10OD018509, S10OD016432, and P41GM066354.

(3) In your Data Availability statement, you have not specified where the minimal data set underlying the results described in your manuscript can be found.

The minimal data set, including NMR data and potentiometric titration data, has been uploaded as supplemental information.

Reviewer 1:

(1) Only one minor issue - a relict from a previous submission: "The reduction potential of Safranine O at pH 7.0 was first reported by Prince et al. *in this journal* in 1981 to be −280 mV vs the Standard Hydrogen Electrode (SHE) as determined by polarography [9]"

The offending verbiage has been removed. The sentence now reads: "The reduction potential of Safranine O at pH 7.0 was first reported by Prince et al. in 1981 to be −280 mV vs the Standard Hydrogen Electrode (SHE) as determined by polarography [9]." We thank the reviewer for catching our embarrassing error.

<u>Reviewer 2:</u>

(1) Page 8, second line from bottom – random use of italics.

If we've found the correct area, these italics are meant to indicate the atom type being discussed in NMR assignments. This is a common usage in the organic literature.

(2) Page 9, line 9 – the authors write "in this journal in 1981". Does this betray a previous, unsuccessful submission to BBA?

The offending verbiage has been removed. The sentence now reads: "The reduction potential of Safranine O at pH 7.0 was first reported by Prince et al. in 1981 to be −280 mV vs the Standard Hydrogen Electrode (SHE) as determined by polarography [9]." We thank the reviewer for catching our embarrassing error.

(3) Page 14, Figure legend. Some additional punctuation would be helpful here.

A number of missing periods were added. We thank the reviewer for catching this.

(4) Page 14, last line and Page 15, first line - what does "Figure 3c and 3d" refer to here?

This verbiage was left over from a previous version of the figure. The sentence now reads: "The major HPLC component, **II**, displays ^{1}H and ^{13}C-spectra characteristic of a molecule with C2 symmetry (Figure 3), and can be readily identified as authentic safranine O." We thank the reviewer for catching this.

Alterations in the manuscript are highlighted in yellow. We thank you for your time and attention, and hope these changes make a convincing argument for publication.

Best Regards,

Ronald L. Koder, Ph.D.

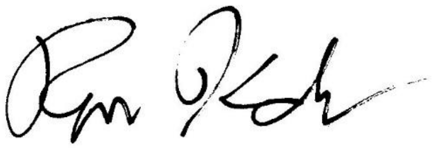

Supplement: Attachment — Submitted filename: Response to reviewers.pdf [file pone.0265105.s003.pdf]
